# Variation in terpenoids and antioxidant activity of loquat flowers during post-harvest processing: a metabolomics study

**DOI:** 10.3389/fpls.2025.1728193

**Published:** 2026-01-06

**Authors:** Mingzheng Duan, Jinghan Feng, Na Gu, Qinxin Tao, Xue Wang, Jing Li, Zengqun Liu, Xiande Duan, Tongfa Wan, Muhammad Junaid Rao

**Affiliations:** 1 Advanced Institute of Ecological Agriculture and Biodiversity on the Yunnan-Guizhou Plateau, Zhaotong University, Zhaotong, China; 2 Yongshan County Bureau of Agriculture and Rural Affairs, Zhaotong, China; 3 State Key Laboratory for Development and Utilization of Forest Food Resources, Zhejiang Agriculture and Forestry (A&F) University, Hangzhou, China

**Keywords:** DPPH antioxidant activity, freeze-drying, hot-water extraction, loquat flower (Eriobotrya japonica), post-harvest processing, terpenoids

## Abstract

Loquat (*Eriobotrya japonica*) flowers are an underutilized resource rich in bioactive compounds, yet the impact of post-harvest processing on their chemical composition remains poorly understood. This study employed a widely targeted metabolomics approach using UPLC-MS/MS with electrospray ionization in both positive and negative modes (ESI+/ESI-) to investigate how different processing methods, including fresh samples (FS), heat-drying (HD), freeze-drying (FD), and subsequent hot-water extraction (FSP, HDP, FDP), affect the terpenoid profile and antioxidant capacity of loquat flowers. A total of 193 terpenoids were identified. Freeze-drying best preserved the native terpenoid diversity and yielded the highest total content, whereas heat-drying led to significant alterations. Multivariate analyses (PCA, HCA) revealed that the hot-water extraction process itself was the dominant factor, inducing a more profound shift in the terpenoid profile than the initial drying method. This was evidenced by terpenoid content reductions ranging from 59% to 70.7% in the hot-water extracts compared to their raw materials. Accordingly, the highest antioxidant activity was found in the freeze-dried sample (469.38 μg Trolox per gram dry weight) but was markedly reduced in all hot-water extracts (143.39-189.54 μg Trolox per gram extract powder). Thus, processing critically defines product quality, with freeze-drying recommended for terpenoid preservation, while extraction optimization is essential to harness the maximum nutraceutical potential of loquat flowers.

## 1 Introduction

Loquat (*Eriobotrya japonica* Lindl.), a plant of the Rosaceae family, has been cultivated for centuries in China and other subtropical regions, primarily for its succulent fruit (Dhiman et al., 2022). However, traditional medicine has long utilized other parts of the tree, including the flowers, for their therapeutic properties in treating conditions such as chronic bronchitis and inflammation (Zhao, 2024). While previous phytochemical investigations of loquat flowers have identified important constituents like flavonoids and megastigmane glycosides, the comprehensive profile of its terpenoid content remains largely unexplored (Zheng et al., 2018; Shah et al., 2023; Kumar et al., 2024). Given that terpenoids are major contributors to both bioactivity and sensory characteristics (Lu et al., 2016), a systematic characterization of this chemical class in loquat flowers is essential to fully understand and valorize this underutilized botanical resource.

Terpenoids, constituting one of the most extensive and structurally diverse families of natural products, are integral to plant physiology and human health (Mabou and Yossa, 2021). Synthesized from isopentenyl diphosphate and its isomer dimethylallyl diphosphate, these compounds are classified by the number of five-carbon isoprene units into monoterpenoids (C10), sesquiterpenoids (C15), diterpenoids (C20), triterpenoids (C30), and beyond. Beyond their fundamental roles in plants, including attracting pollinators and providing defense mechanisms, terpenoids are paramount to the food and fragrance industries due to their distinctive aromatic profiles (Ninkuu et al., 2021). More critically, a growing body of evidence underscores their significant pharmacological and nutraceutical potential, exhibiting a wide spectrum of bioactivities that include anti-inflammatory, antimicrobial, and anticancer properties (Indumathi et al., 2014). A key mechanism underpinning many of these health benefits is their potent antioxidant activity (Hammed et al., 2025; Rao and Zheng, 2025; Rao et al., 2025). Numerous terpenoids, particularly triterpenoids like ursolic and oleanolic acid, effectively scavenge free radicals, including the stable 2, 2-diphenyl-1-picrylhydrazyl (DPPH) radical, thereby mitigating oxidative stress, a fundamental pathway in aging and chronic diseases (Gudoityte et al., 2021; Nguyen et al., 2021; Khwaza and Aderibigbe, 2024; Günther and Bednarczyk-Cwynar, 2025). This antioxidant capacity makes them highly valuable for developing functional foods and preserving food quality by inhibiting lipid oxidation.

In the context of food science and technology, the journey from raw agricultural material to a stable, consumable product is critically dependent on post-harvest processing techniques (Gao et al., 2021). These methods are designed to ensure safety, extend shelf-life, and ideally, preserve or even enhance the bioactive compound profile. Drying is a fundamental first step, with heat-drying (HD) and freeze-drying (FD) being two prevalent industrial methods. Heat-drying, while cost-effective and efficient, involves elevated temperatures that can degrade thermolabile compounds, including many terpenoids and antioxidants (Zheng et al., 2015, 2018). In contrast, freeze-drying, which removes water via sublimation under vacuum, is renowned for better preserving the structural integrity and volatility of sensitive metabolites. Subsequently, for applications such as herbal tea production, a hot-water extraction is often employed (Duan et al., 2025b, 2025a). This step presents a critical control point, as it can lead to the incomplete extraction of lipophilic compounds, thermal degradation, or chemical transformations, ultimately defining the final composition of the extract consumed (Prado and Rostagno, 2022; Ramesh et al., 2024).

Despite the recognized importance of terpenoids and the potential of loquat flowers, a significant knowledge gap exists at the intersection of food processing and phytochemistry. Previous phytochemical studies on loquat have primarily focused on other plant parts or compound classes (Zhao, 2024). For instance, research has extensively documented the flavonoid profiles and antioxidant activities of loquat leaves and fruits (Kumar et al., 2024; Duan et al., 2025a). Recent studies indicate the growing interest in loquat flowers, from optimizing their processing into tea (Zheng et al., 2015) to maximizing the extraction of their health-promoting flavonoids (Duan et al., 2025b). However, these studies have not provided a comprehensive characterization of the terpenoid metabolome.

Therefore, this study was designed to bridge this critical gap by employing a widely targeted LC-MS/MS-based metabolomics approach coupled with biochemical assays. The specific objectives were to: (1) comprehensively profile the terpenoid compounds in loquat flowers; (2) evaluate the impact of different processing methods (fresh, heat-dried, freeze-dried) and subsequent hot-water extraction on the terpenoid content and composition; and (3) assess the consequent changes in the overall antioxidant potential using the DPPH radical scavenging assay. By elucidating the relationship between processing technology and the terpenoid-antioxidant landscape, this study provides a scientific foundation for optimizing processing strategies to maximize the value of loquat flowers in functional food and nutraceutical applications.

## 2 Methods

### 2.1 Harvesting of loquat flowers

Loquat (*Eriobotrya japonica* cv. Dawuxing) flowers were harvested on November 25, 2024, from Xiluodu Town, Yongshan County (103.64°E, 28.24°N), Zhaotong City, Yunnan Province, China. To ensure uniformity in developmental stage and physiological condition, flowers were collected at the partially bloomed bud phase. Samples were obtained from six healthy, mature plants cultivated under comparable environmental conditions. Harvesting was conducted using sterilized scissors, with careful handling to preserve floral integrity. The collected specimens were immediately placed in sterile containers to minimize contamination and mechanical damage during transport.

### 2.2 Laboratory processing of flowers

In the laboratory, samples underwent a purification process involving immersion in deionized water with gentle agitation to eliminate surface impurities. Residual moisture was removed using sterile absorbent material, followed by air-drying under ambient conditions. The processed flowers were then divided into three preservation treatments: refrigeration at 4 °C (Fresh samples, FS), thermal dehydration (Heat drying, HD) dehydration at 60 °C for 6 h until complete desiccation, a moderate-temperature protocol chosen to achieve efficient drying while limiting thermal degradation; and lyophilization (Fresh drying, FD) (initial freezing at −20 °C followed by vacuum drying at −50 °C for 48 h).

### 2.3 Hot-water extraction procedure

All raw samples (FS, HD, and FD) underwent aqueous extraction, using a thermal (FSP, HDP, and FDP) method at 90 °C for 30 min with a fixed biomass-to-solvent ratio of 1:20 (w/v). Following extraction, the mixture was subjected to gravity separation at room temperature for 6 h to isolate the supernatant. The resulting extract was subsequently freeze-dried, involving preliminary freezing at −40°C followed by vacuum dehydration for 48 h, to obtain a stable powdered form suitable for long-term storage.

Strict quality control measures were applied throughout the experimental workflow, including the use of precision-controlled drying and lyophilization equipment, ultra-low temperature storage systems, and aseptic handling techniques to preserve sample integrity. The final extracts. fresh, thermally dehydrated, and lyophilized, were hermetically sealed, properly labeled, and stored for subsequent phytochemical investigations, with a particular focus on terpenoid profiling.

### 2.4 Terpenoid profiling by UPLC–MS/MS

Terpenoid composition in loquat flowers (expressed on a dry weight basis) was analyzed using ultra-performance liquid chromatography coupled with tandem mass spectrometry (UPLC–MS/MS) following established protocols (Chen et al., 2013). The floral material was first lyophilized for 63 hours and subsequently ground into a fine powder at 30 Hz for 1.5 minutes. For metabolite extraction, 30 mg of powdered tissue was suspended in 1.5 mL of 70% methanol containing an internal standards (2-chlorophenylalanine, CAS 14091-11-3, at a concentration of 1 µg/mL) for quality control, and the mixture was maintained at −20 °C. Samples were intermittently vortexed (six cycles of 30 seconds each) and centrifuged at 12, 000 rpm for 3 minutes. The resulting supernatants were filtered through 0.22 μm membranes prior to instrumental analysis.

Chromatographic separation was performed on an Agilent SB-C18 column (2.1 × 100 mm, 1.8 μm particle size) using a binary gradient system composed of 0.1% formic acid in water (mobile phase A) and acetonitrile (mobile phase B). The gradient was programmed to increase from 5% to 95% B within 9 minutes at a flow rate of 0.35 mL/min, with the column maintained at 40 °C. Terpenoid annotation was performed by the service provider (MetWare, Wuhan, China) using a widely targeted metabolomics approach based on their customized database (MWDB). A three-level confidence framework was applied: Level 1 (confirmation with authentic standards), Level 2 (spectral match scores between 0.5–0.7), and Level 3 (annotation based on precursor/product ion pairs and consistent chromatographic behavior). **Supplementary Table S1** provides the detailed identification parameters for all detected terpenoids, including compound name, class, formula, precursor ion (Q1), product ion (Q3), molecular weight, ionization mode, confidence level, and CAS number where available. This tiered approach, supported by the fundamental mass spectrometry data provided, ensured robust compound identification in accordance with previously validated methodologies (Chen et al., 2013).

### 2.5 DPPH assay to evaluate loquat flowers free radical scavenging activity

The antioxidant potential of loquat flowers subjected to different processing methods was evaluated through the 2, 2-diphenyl-1-picrylhydrazyl (DPPH) radical scavenging assay, following the procedure described by Dudonne et al (Dudonne et al., 2009). The experimental materials included fresh flowers, freeze-dried samples, oven-dried samples, and their respective hot-water-extracted powders.

For sample preparation, 0.1 g of each specimen was homogenized in 1 mL of 80% methanol. The mixtures were then subjected to ultrasonic-assisted extraction at 60 °C for 30 minutes with intermittent agitation, after which they were centrifuged at 12, 000 rpm to collect the supernatant. The assay was performed in two sets for each sample: (1) the test mixture, combining equal volumes (150 µL) of the supernatant extract with the DPPH working solution (prepared in ethanol), and (2) a sample blank, combining 150 µL of the extract with 150 µL of ethanol to correct for the sample’s inherent absorbance. A control containing 150 µL of methanol and 150 µL of the DPPH working solution was also included. Following a 30-minute incubation in the dark, absorbance was measured at 517 nm using a microplate reader. The absorbance of the sample blank was subtracted from the absorbance of the corresponding test mixture before calculating the radical scavenging activity.

The antioxidant capacity of the samples was expressed as micrograms of Trolox equivalents per gram of sample (µg TE/g): per gram dry weight for raw materials (FS, HD, FD) and per gram of the final extract powder for hot-water extracts (FSP, HDP, FDP), based on calibration against a standard curve constructed with Trolox concentrations ranging from 0 to 25 µg/mL.

### 2.6 Bioinformatic and statistical analysis

To investigate the distribution of terpenoids in different processed material of loquat flower, multivariate statistical approaches were applied. Prior to analysis, the metabolomic datasets were standardized using Z-score normalization. To enhance group separation while minimizing within-group variation, hierarchical cluster analysis (HCA) and principal component analysis (PCA) were performed through the OmicShare online analytical platform (Mu et al., 2024). Graphical representations and error estimations were generated using Microsoft Excel (Redmond, WA, USA). For the statistical assessment of biochemical traits, Statistix 8.1 software (Tallahassee, FL, USA) was utilized, with all experiments conducted in triplicate. Upon obtaining a significant F-test, the Fisher’s Least Significant Difference (LSD) test was employed to evaluate specific differences in antioxidant activity and terpenoid levels among loquat samples at a significance level of p < 0.05. Variable importance in projection (VIP) scores was calculated using orthogonal partial least squares-discriminant analysis (OPLS-DA) implemented in the MetaboAnalystR package within the R (Thévenot et al., 2015; Chong and Xia, 2018). The OPLS-DA model was validated using a permutation test (200 permutations) to guard against overfitting. Models with a Q2 value (goodness of prediction) greater than 0.5 from the permutation test were considered statistically valid and robust. Metabolites with VIP values greater than 1.0 were considered key discriminatory biomarkers.

Significantly altered metabolites were identified using a dual-criteria strategy: statistical significance was determined by Student’s t-test with false discovery rate (FDR) correction (p < 0.05) (Colquhoun, 2014), while biological relevance was defined by fold-change thresholds (|log2FC| ≥ 1, equivalent to FC ≥ 2 or ≤ 0.5). Overlapping metabolites across groups were visualized using the EVenn web-based tool (https://www.bic.ac.cn/EVenn/#/) (Mu et al., 2024).

## 3 Results

A widely targeted LC-MS/MS-based metabolomics approach identified 193 terpenoid compounds in loquat flowers under different processing conditions. These terpenoids included 81 triterpenes (non-saponin), 60 sesquiterpenoids, 22 monoterpenoids, 15 triterpene saponins, 14 diterpenoids, and one terpene (**Supplementary Table S1**), confirming that all 193 compounds are unique entities with no overlap between categories. Detailed information for all identified compounds, including chemical formulas, precursor and product ion masses (Q1, Q3), molecular weight, ionization mode, CAS number, and KEGG compound ID, is provided in **Supplementary Table S1**.

### 3.1 Processing methods significantly alter terpenoid content and profile similarity

**Figure 1:**
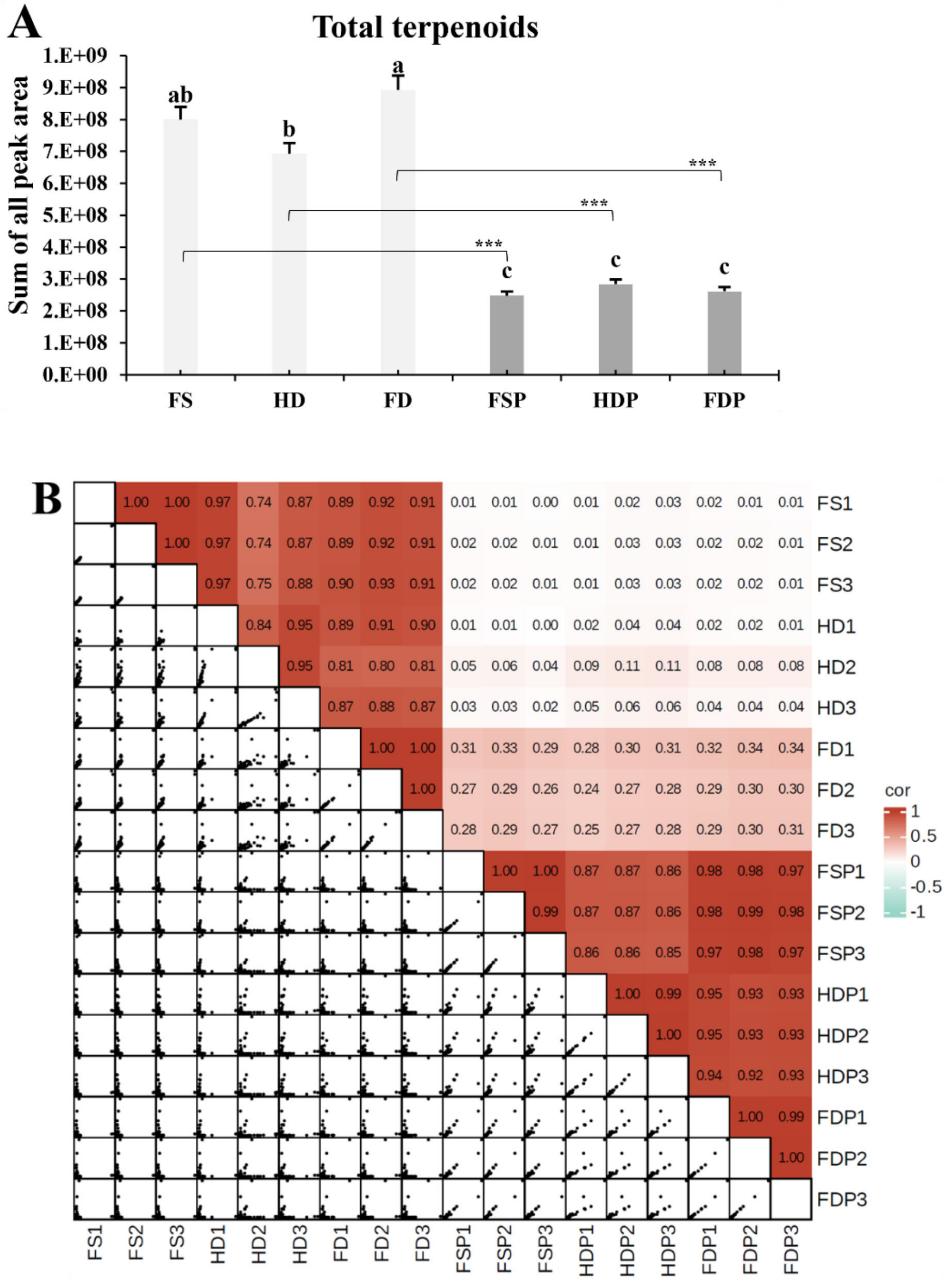
Impact of processing methods on the terpenoid profile of loquat flowers. **(A)** Total terpenoid content, determined as the sum of peak areas for all identified terpenoids. Data are presented as mean ± SD (n=3). Different lowercase letters (a-c) above bars indicate significant differences according to Fisher’s LSD test (P < 0.05) within raw materials or within extracts. Asterisks (***) indicate significant differences (p < 0.001) between each raw material and its corresponding extract. **(B)** Pearson correlation matrix illustrating the similarity of terpenoid profiles between samples. The color scale and numerical values represent the correlation coefficient (r), where values approaching 1.0 (red) indicate high similarity and values near 0 (white/pale red) indicate low similarity. FS, fresh sample; HD, heat-dried; FD, freeze-dried; FSP, fresh sample hot-water extract; HDP, heat-dried sample hot-water extract; FDP, freeze-dried sample hot-water extract.

The total terpenoid content, determined as the cumulative peak area of all identified compounds, was significantly influenced by the processing method (**Figure 1A**). Among the raw materials, the freeze-dried (FD) sample exhibited the highest total terpenoid content, followed by the fresh (FS) and heat-dried (HD) samples. This suggests that freeze-drying is a more effective method for preserving the overall terpenoid pool compared to heat-drying, which likely induces thermal degradation or volatilization of these compounds. Conversely, a substantial reduction in total terpenoid content was observed across all hot-water extracts (FSP, HDP, FDP) compared to their respective raw materials (**Figure 1A**). Statistical analysis confirmed extremely significant differences between each raw material and its corresponding extract (p < 0.001 for FS vs FSP, HD vs HDP, and FD vs FDP). The hot-water extraction process resulted in substantial terpenoid losses, with the fresh sample extract (FSP) losing 69.0%, the heat-dried extract (HDP) losing 59.0%, and the freeze-dried extract (FDP) losing 70.7% of their total terpenoid content compared to the unextracted materials. This indicates that the extraction process itself, while transferring terpenoids into the aqueous phase, does so incompletely, leaving a significant portion unextracted or potentially leading to compound degradation under hot-water conditions. Notably, the hot-water extracts showed a hierarchy of terpenoid retention, with the heat-dried extract (HDP) containing the highest content, followed by the freeze-dried extract (FDP), and then the fresh sample extract (FSP) containing the lowest. This demonstrates that the post-harvest processing technique significantly impacts the extractability and stability of these compounds.

The Pearson correlation matrix revealed strong intra-group similarities and pronounced inter-group differences (**Figure 1B**). Very high correlation coefficients (r > 0.95) were observed among biological replicates within each processing group, confirming the high reproducibility of the analytical method. Crucially, the strong positive correlation between FS and FD (r ~ 0.89-0.91) indicates that freeze-drying effectively preserves the native terpenoid profile of fresh flowers. In stark contrast, the correlations between all raw materials (FS, HD, FD) and their respective hot-water extracts (FSP, HDP, FDP) were markedly low (r < 0.31), demonstrating that the extraction process induces a fundamental alteration of the terpenoid profile (**Figure 1B**). This pattern correlates with the substantial terpenoid reduction observed in **Figure 1A**. Furthermore, the hot-water extracts (FSP, HDP, FDP) formed highly correlated clusters with each other (r ~ 0.86-0.97). This indicates that both drying methods and their hot-water extracts cause significant changes compared to the fresh state, they each generate a distinct and reproducible terpenoid profile.

### 3.2 Hierarchical cluster analysis reveals distinct grouping of terpenoids by processing method

**Figure 2:**
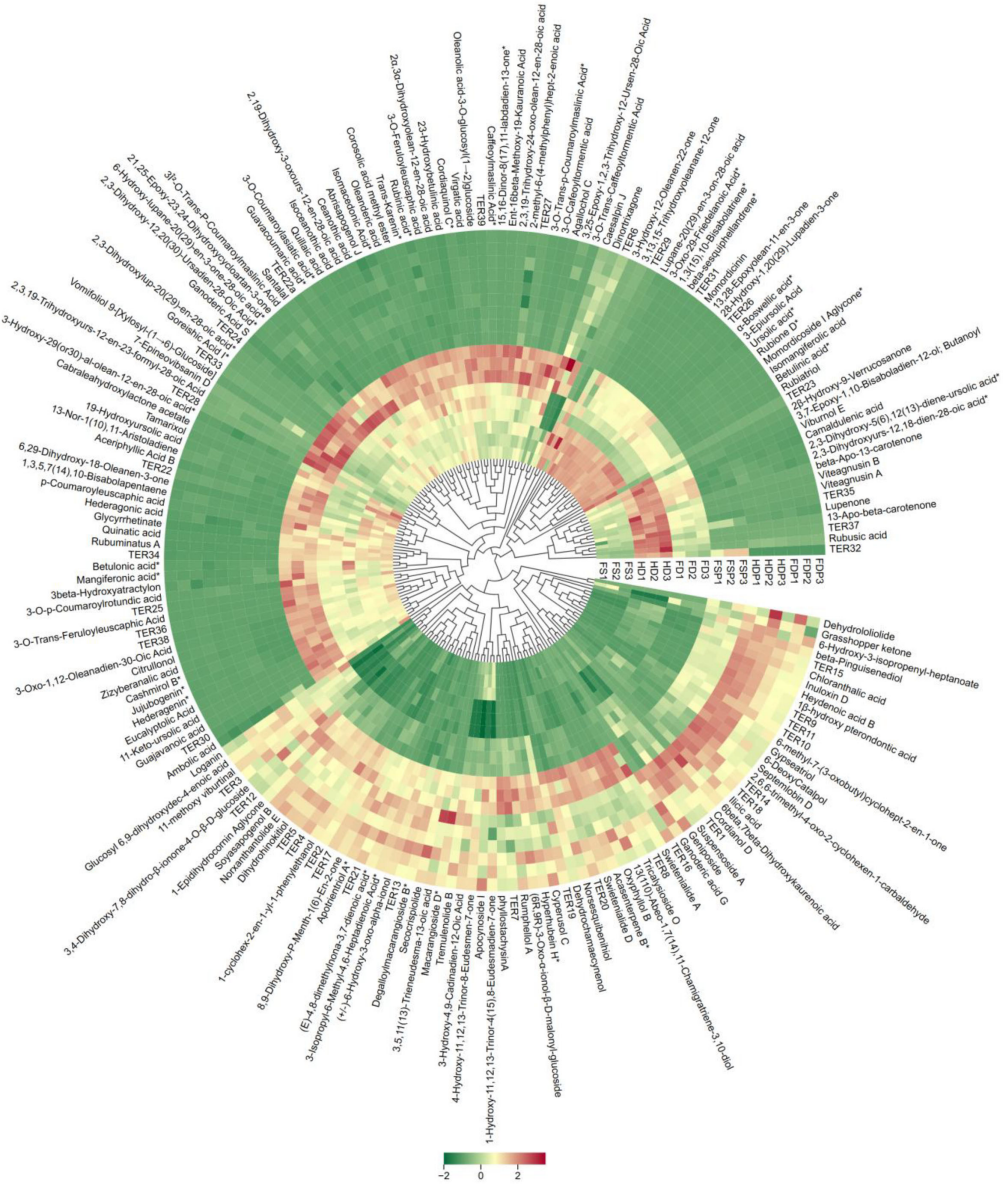
Hierarchical cluster analysis (HCA) of terpenoid profiles from loquat flowers subjected to different processing methods (TER1-TER39 corresponds to individual terpenoid characterized in **Supplementary Table S2**). The rows were z-score normalized and the heatmap visualizes relative terpenoid abundance, where red indicates high abundance and green indicates low abundance. FS, fresh sample; HD, heat-dried; FD, freeze-dried; FSP, fresh sample hot-water extract; HDP, heat-dried sample hot-water extract; FDP, freeze-dried sample hot-water extract.

Hierarchical cluster analysis (HCA) was performed to evaluate the similarity of terpenoid profiles among the differently processed loquat flower samples (**Figure 2**). The resulting dendrogram exhibited a clear divergence, forming two primary clusters. The first cluster comprised exclusively the raw materials (FS, HD, FD), while the second cluster contained all the hot-water extracts (FSP, HDP, and FDP), indicating a high degree of dissimilarity in terpenoid composition between raw materials and their extracts. HCA revealed that compounds such as 11-methoxy viburtinal and Swietenialide D were found in higher abundance in the hot-water extract groups. Conversely, terpenoids including 28-Hydroxy-1, 20(29)-Lupadien-3-one, 13, 28-Epoxyolean-11-en-3-one, and Rubione D were predominantly more abundant in the FS, HD, FD group than their corresponding hot-water extracts, indicating a variable extraction efficiency or potential thermal transformation during processing (**Figure 2**). These results demonstrate that each processing method imparts a unique and characteristic chemical signature on the terpenoids, that is more significant than the differences between each specific processing techniques or the subsequent extraction step.

### 3.3 PCA reveals distinct grouping of samples by processing method

**Figure 3:**
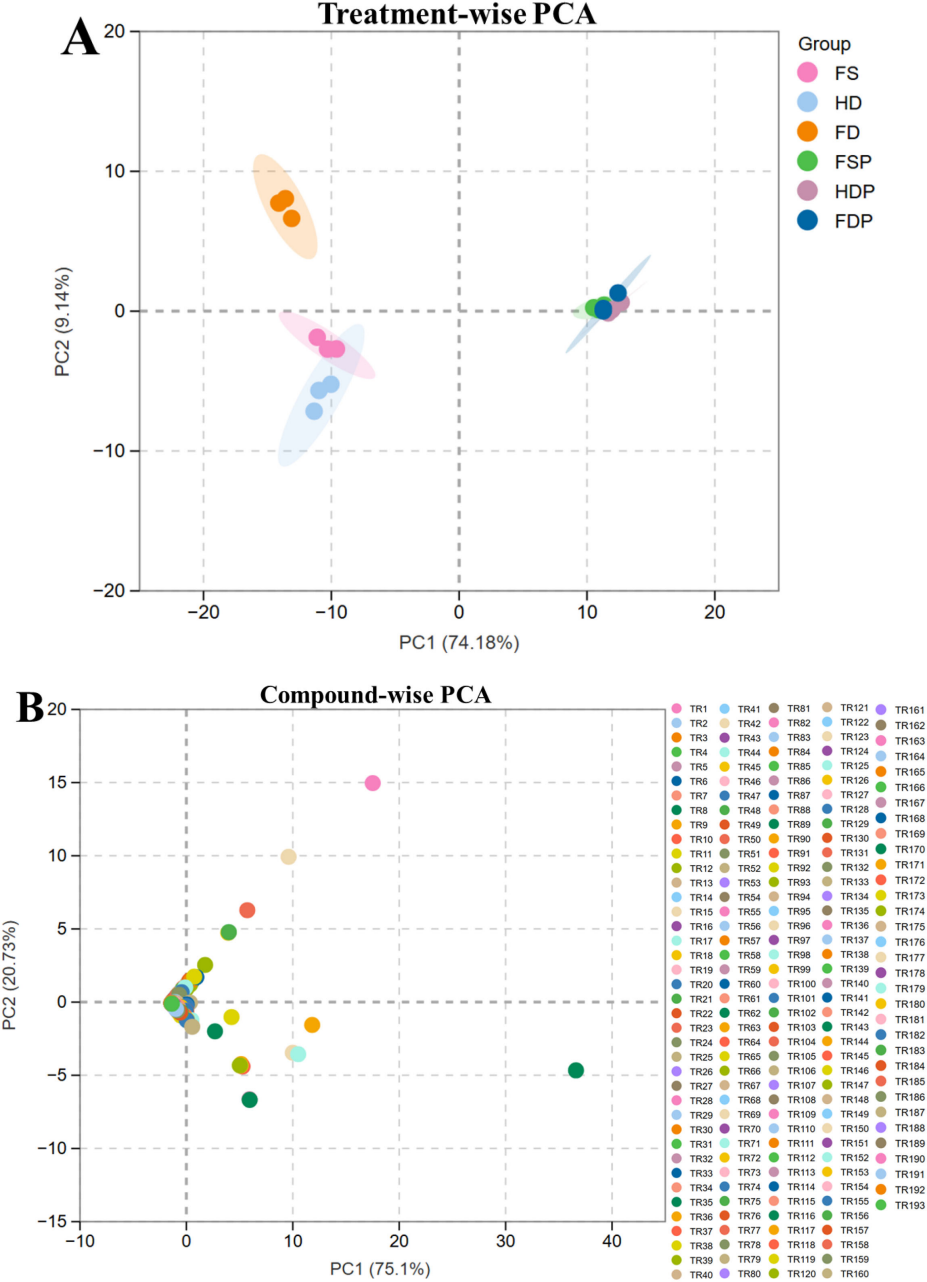
Principal component analysis (PCA) score plot of the terpenoid profiles from loquat flowers subjected to different processing methods. **(A)** Treatment/processing method-wise PCA; **(B)** Terpenoid compounds-wise PCA (TR1-TR193 corresponds to individual terpenoid represented in **Supplementary Table S2**). FS, fresh sample; HD, heat-dried; FD, freeze-dried; FSP, fresh sample hot-water extract; HDP, heat-dried sample hot-water extract; FDP, freeze-dried sample hot-water extract.

Principal component analysis (PCA) was employed to visualize the variations in the terpenoid profiles among the different loquat flower materials and their processed extracts. The score plot (**Figure 3A**) revealed a clear separation along the first principal component (PC1) and second PC2, which accounted for 74.18% and 9.14% of the total variance, respectively (**Figure 3A**). The analysis revealed three distinct groupings: (1) the fresh (FS) and heat-dried (HD) samples clustered closely together in the negative PC1 region, indicating high similarity in their terpenoid composition; (2) the freeze-dried (FD) samples formed a separate group; and (3) all hot-water extracts (FSP, HDP, FDP) clustered together in the positive PC1 region, clearly separated from the raw materials. This demonstrates that both drying method and extraction process significantly influence the terpenoid profile. The compounds-wise PCA of the terpenoid profiles showed that the first two principal components captured a high cumulative variance of 95.83%, with PC1 and PC2 accounting for 75.1% and 20.73% of the total variance, respectively (**Figure 3B**). The PCA plot reveals a distinct spatial distribution of the terpenoid compounds (TR1-TR193), suggesting that specific terpenoids are characteristic of sample types (**Figure 3B**). This pronounced separation demonstrates that the drying process, irrespective of the method (thermal or freeze-drying), induces a significant and substantial alteration in the terpenoid profile compared to the fresh state. The PCA results corroborate with the HCA findings, confirming that processing method is the dominant factor influencing the terpenoid composition of loquat flowers.

### 3.3 Impact of drying and extraction methods on terpenoid composition

The quantitative analysis of major terpenoids, selected based on their highest relative abundance across all samples (representing the most prevalent compounds detected in loquat flowers), revealed substantial variation influenced by both drying and extraction processes. Fresh samples (FS) exhibited high levels of certain compounds such as 13, 28-Epoxyolean-11-en-3-one and 28-Hydroxy-1, 20(29)-Lupadien-3-one. Heat-drying (HD) led to a marked increase in compounds like Viteagnusin B and Heydenoic acid B, while freeze-drying (FD) better preserved compounds such as ceanothic acid and virgatic acid, showing levels comparable to or exceeding those in FS. Hot-water extraction generally reduced the abundance of most terpenoids across all sample types (FSP, HDP, FDP), with particularly notable declines in terpenoids such as Septemlobin D and Swietenialide D. However, HDP extracts retained relatively higher concentrations of several terpenoids compared to FDP, suggesting that heat-dried material may yield a more terpenoid-rich extract. These results underscore the critical impact of post-harvest processing on the preservation and extractability of bioactive terpenoids in loquat flowers.

### 3.4 Extraction process is the primary determinant of terpenoid profile over drying method

**Figure 4:**
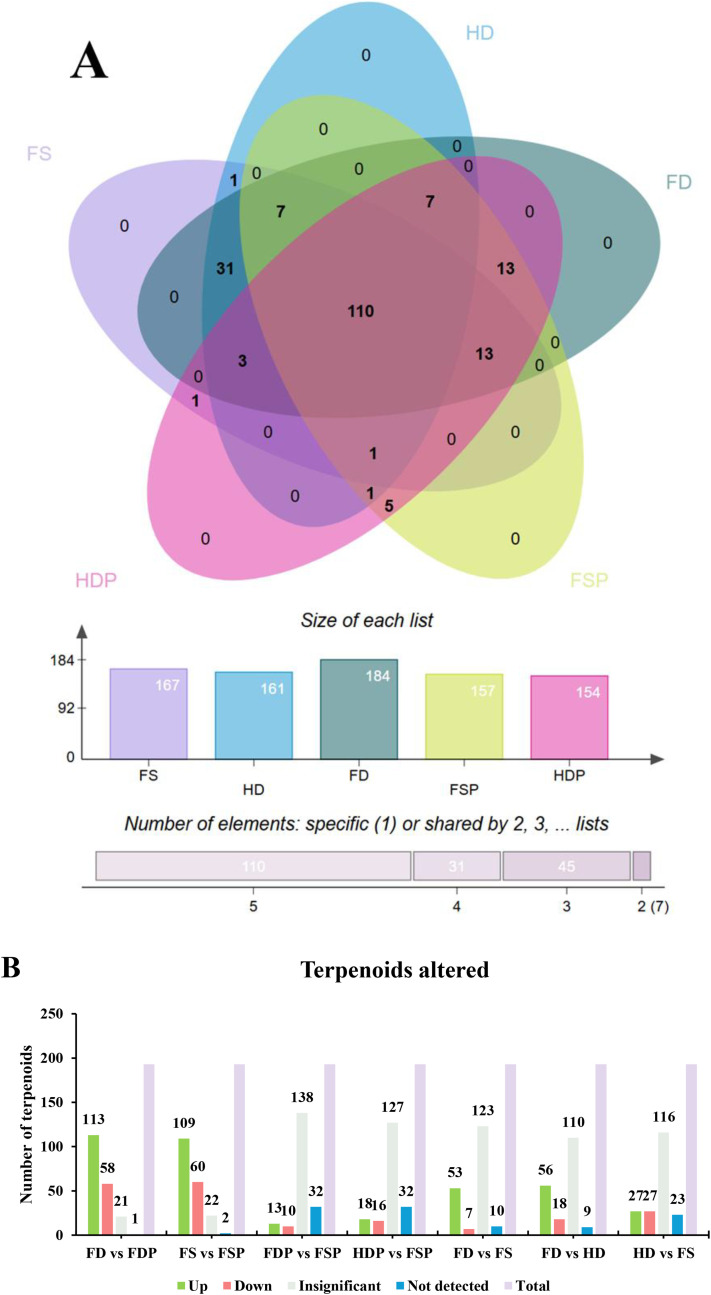
Analysis of terpenoid abundance alteration in loquat flowers under different processing conditions. **(A)** Five-set Venn diagram showing the distribution of terpenoids across fresh (FS, purple), heat-dried (HD, blue), freeze-dried (FD, teal) samples and their hot-water extracts (FSP, yellow; HDP, pink). The accompanying bar chart quantifies the number of compounds shared across multiple sample groups. **(B)** Bar chart classifying 193 terpenoids as significantly upregulated (Up; Log2FC > 1, VIP > 1), downregulated (Down; Log2FC < -1, VIP > 1), insignificant, or not detected in pairwise comparisons between sample types (**Supplementary Table S3**). FS, fresh sample; HD, heat-dried; FD, freeze-dried; FSP, fresh sample hot-water extract; HDP, heat-dried sample hot-water extract; FDP, freeze-dried sample hot-water extract.

The Venn diagram analysis (**Figure 4A**) provides crucial insights into how post-harvest processing (fresh, heat-drying, freeze-drying) and subsequent hot-water extraction influence the terpenoid profile of loquat flowers, which are key to their aromatic and potential bioactive properties. A core of 110 terpenoids was consistently present across all samples, forming the fundamental flavor base. Freeze-drying best preserved the terpenoid diversity, with the highest total number of detectable terpenoids (184 compounds including110 core metabolites plus 74 additional compounds preserved by freeze-drying), which suggests minimal compound degradation (**Figure 4A**). In contrast, heat-drying and the extraction process led to a reduction in the number of detectable terpenoids in the final extracts (HDP: 154; FSP: 157). The distinct patterns of shared terpenoids, such as the 31 compounds common only to the three (FS, HD, FD) types, are likely volatiles affected by the extraction process.

The differential abundance analysis of terpenoids revealed that the transition from raw materials to their hot-water extracts (FD vs FDP and FS vs FSP) involved the most substantial metabolic shifts, with a high number of compounds being significantly upregulated (113 and 109, respectively) or downregulated (58 and 60, respectively) (**Figure 4B**). In contrast, comparisons between the different extracts (FDP vs FSP, HDP vs FSP) and between the raw drying methods (FD vs FS, FD vs HD, HD vs FS) showed a majority of terpenoids with insignificant changes or that were not detected, indicating that the extraction process itself is a greater source of variation in terpenoid profile than the specific drying technique applied to the raw material.

### 3.5 Significant alterations of key terpenoids by different processing techniques

**Figure 5:**
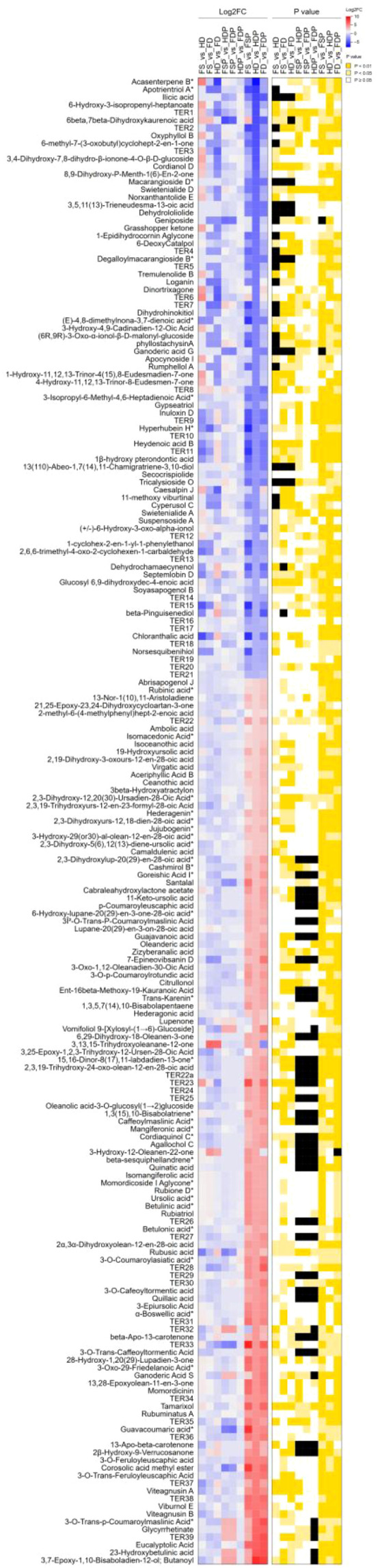
Heatmap illustrates the log2-fold change (Log2FC) of 193 terpenoid compounds across different processing methods of loquat flowers. Comparisons include FS, fresh sample; HD, heat-dried; FD, freeze-dried; FSP, fresh sample hot-water extract; HDP, heat-dried sample hot-water extract; FDP, freeze-dried sample hot-water extract. Each row represents a terpenoid metabolite, with color intensity indicating the magnitude and direction of Log2FC (blue for downregulation, red for upregulation). Statistical significance (p < 0.05) for each comparison is provided in **Supplementary Table S3**. Compounds marked with an asterisk (*) denote isomers. The data highlight the impact of drying and extraction methods on terpenoid composition.

The heatmap reveals significant variation (Log2FC > 1, VIP > 1) in terpenoid abundance and regulation across processing conditions (**Supplementary Table S3**; **Figure 5**). Multiple terpenoids, including ursolic acid, betulinic acid, and their derivatives, show pronounced upregulation in freeze- and heat-dried samples (FD and HD) compared to their hot-water extracts (FDP and HDP). Freeze-drying (FD and FDP) generally preserved a terpenoid profile closer to fresh samples, though distinct compositional shifts were still evident. Several isomers, such as hederagenin and mangiferolic acid, exhibited differential responses to processing (**Figure 5**), underscoring the sensitivity of terpenoid stability to thermal treatment. Analysis of specific compounds revealed that HDP extracts retained relatively higher concentrations of several individual terpenoids compared to FDP, suggesting that heat-dried material may yield an extract enriched in certain bioactive terpenoids, despite the slightly lower total terpenoid content shown in **Figure 1A**.

The targeted analysis identified 50 terpenoids that underwent significant alteration (Log2FC > 3.5, VIP > 1.1, P-value < 0.05) across different processing comparisons (**Table 1**). The transition from freeze-dried material to its hot-water extract (FDP vs FD) represented the most significant shift, with 24 compounds significantly (Log2FC > ± 3.5) altered (**Table 1**). Notably, compounds such as beta-Pinguisenediol, Ilicic acid, and 6beta, 7beta-Dihydroxykaurenoic acid were markedly upregulated in the extract, while several complex, hydroxylated ursane and lupane-type triterpenoid acids (e.g., 3-O-Trans-p-Coumaroylmaslinic Acid) were strongly downregulated (Log2FC > -3.5). Furthermore, key comparisons between drying methods (FD vs HD, FD vs FS) highlighted specific terpenoids like Apotrientriol A and Loganin as being highly responsive to thermal treatment. The compound 6beta, 7beta-Dihydroxykaurenoic acid was consistently upregulated in extract comparisons (FDP vs FSP, HDP vs FSP), underscoring its extractability and stability, positioning it as a critical marker influenced by processing.

**Table 1:** Significantly altered terpenoids (Log2FC > 3.5, VIP > 1.1, P-value < 0.05) in key pairwise comparisons of loquat flower processing methods.

Serial No.	Compounds	FD vs HD_				
		VIP	P-value	Fold_Change	Log2FC	Type
1	Acasenterpene B*	1.16	0.020	55.76	5.80	up
2	(1S, 6S, 9R)-6, 10, 10-trimethyl-2-methylidenebicyclo[7.2.0]undecan-5-one*	1.16	0.005	26.15	4.71	up
3	Caesalpin J	1.16	0.006	22.61	4.50	up
4	Dinortrixagone	1.14	0.094	21.88	4.45	up
5	1-(4-hydroxy-pentyl)-2, 8a-dimethyl-5-methylene-decahydro-naphthalene-2, 6-diol	1.17	0.001	20.92	4.39	up
6	ent-3α, 16β, 17, 19-Tetrahydroxykauran-19-ylacetate-17-O-β-D-glucopyranoside	1.17	0.000	14.95	3.90	up
7	Apotrientriol A*	1.10	0.105	13.91	3.80	up
8	4-methyl-4-{[3, 4, 5-trihydroxy-6-(hydroxymethyl)oxan-2-yl]oxy}oxan-2-one	1.16	0.009	13.88	3.79	up
9	Loganin	1.15	0.036	12.78	3.68	up
10	3, 13, 15-Trihydroxyoleanane-12-one	1.17	0.004	0.02	-5.68	down
Serial No.	Compounds	FD vs FS				
		VIP	P-value	Fold_Change	Log2FC	Type
11	Hyperhubein H*	1.20	0.029	18.59	4.22	up
12	ent-3α, 16β, 17, 19-Tetrahydroxykauran-19-ylacetate-17-O-β-D-glucopyranoside	1.21	0.000	14.95	3.90	up
13	Apotrientriol A*	1.14	0.105	13.91	3.80	up
14	Loganin	1.20	0.036	12.78	3.68	up
15	3-Hydroxy-12-Oleanen-22-one	1.21	0.013	0.06	-4.02	down
16	3, 13, 15-Trihydroxyoleanane-12-one	1.21	0.002	0.02	-5.84	down
Serial No.	Compounds	FDP vs FD				
		VIP	P-value	Fold_Change	Log2FC	Type
17	beta-Pinguisenediol	1.11	0.005	28.31	4.82	up
18	Ilicic acid	1.10	0.031	27.02	4.76	up
19	6beta, 7beta-Dihydroxykaurenoic acid	1.11	0.004	22.50	4.49	up
20	Dehydrochamaecynenol	1.11	0.002	17.26	4.11	up
21	3-[(Arabinosyl)oxy]-23-hydroxyurs-12, 19(29)-dien-28-oic acid-28-O-glucosyl ester	1.09	0.016	15.14	3.92	up
22	Macarangioside D*	1.11	0.007	14.76	3.88	up
23	Heydenoic acid B	1.11	0.003	14.34	3.84	up
24	(3S, 4aR, 5R, 6S)-6-hydroxy-4a, 5-dimethyl-3-(prop-1-en-2-yl)-4, 4a, 5, 6-tetrahydronaphthalen-2(3H)-one	1.11	0.004	14.28	3.84	up
25	3, 5, 11(13)-Trieneudesma-13-oic acid	1.09	0.058	14.12	3.82	up
26	Inuloxin D	1.11	0.004	13.64	3.77	up
27	Dehydrololiolide	1.05	0.171	12.92	3.69	up
28	Chloranthalic acid	1.09	0.007	12.72	3.67	up
29	5α, 6α, 7β, 10β-11α, 13-dihydro-4(15)-eudesmene-12, 6-olide Tsoongiodendroonolide	1.11	0.005	12.47	3.64	up
30	Degalloylmacarangioside B*	1.10	0.024	12.09	3.60	up
31	1β-hydroxy pterondontic acid	1.11	0.007	11.59	3.53	up
32	13(110)-Abeo-1, 7(14), 11-Chamigratriene-3, 10-diol	1.09	0.055	11.38	3.51	up
33	(E)-4, 8-dimethylnona-3, 7-dienoic acid*	1.10	0.019	11.37	3.51	up
34	2, 3-Dihydroxy-12-Ursen-28-Oic Acid 3-O-(3-Hydroxy-4-Methoxy-E-Cinnamoyl)	1.09	0.023	0.01	-6.22	down
35	5, 8α-dioxy-3β, 16α-dihydroxyl-lanost-7(11), 24-dien-21-oic acid	1.11	0.010	0.01	-6.32	down
36	Eucalyptolic Acid	1.11	0.007	0.01	-6.62	down
37	3, 23-Dihydroxylup-20(29)-en-28-oic acid (23-Hydroxybetulinic acid)	1.11	0.001	0.01	-6.74	down
38	3-O-Trans-p-Coumaroylmaslinic Acid*	1.11	0.016	0.01	-6.78	down
39	2, 3, 23-Trihydroxy-12-Ursen-28-Oic Acid 3-O-(4-Hydroxy-E-Cinnamoyl)*	1.11	0.004	0.01	-6.84	down
40	3, 7-Epoxy-1, 10-Bisaboladien-12-ol; Butanoyl	1.11	0.002	0.01	-7.37	down
Serial No.	Compounds	FDP vs FSP				
		VIP	P-value	Fold_Change	Log2FC	Type
41	6beta, 7beta-Dihydroxykaurenoic acid	1.41	0.004	22.50	4.49	up
42	24-p-Coumaroyloxy-2, 3-dihydroxyurs-12-en-28-oic acid (Guavacoumaric acid)*	1.40	0.019	14.04	3.81	up
43	Geniposide	1.40	0.032	13.49	3.75	up
44	Ganoderic acid G	1.40	0.022	10.90	3.45	up
45	(3S)-3-isopropenyl-6-oxoheptanoate	1.41	0.014	0.08	-3.67	down
Serial No.	Compounds	HDP vs FSP				
		VIP	P-value	Fold_Change	Log2FC	Type
46	6beta, 7beta-Dihydroxykaurenoic acid	1.19	0.029	36.27	5.18	up
47	Geniposide	1.20	0.002	16.21	4.02	up
48	2, 3-Dihydroxyurs-12-en-28-oic acid methyl ester (Corosolic acid methyl ester)	1.17	0.055	12.04	3.59	up
49	24-p-Coumaroyloxy-2, 3-dihydroxyurs-12-en-28-oic acid (Guavacoumaric acid)*	1.13	0.129	11.63	3.54	up
50	(3S)-3-isopropenyl-6-oxoheptanoate	1.19	0.014	0.08	-3.67	down

FS, fresh sample; HD, heat-dried; FD, freeze-dried; FSP, FS hot-water extract; HDP, HD hot-water extract; FDP, FD hot-water extract. * means isomers.

### 3.6 Evaluation of antioxidant activity by DPPH assay

**Figure 6:**
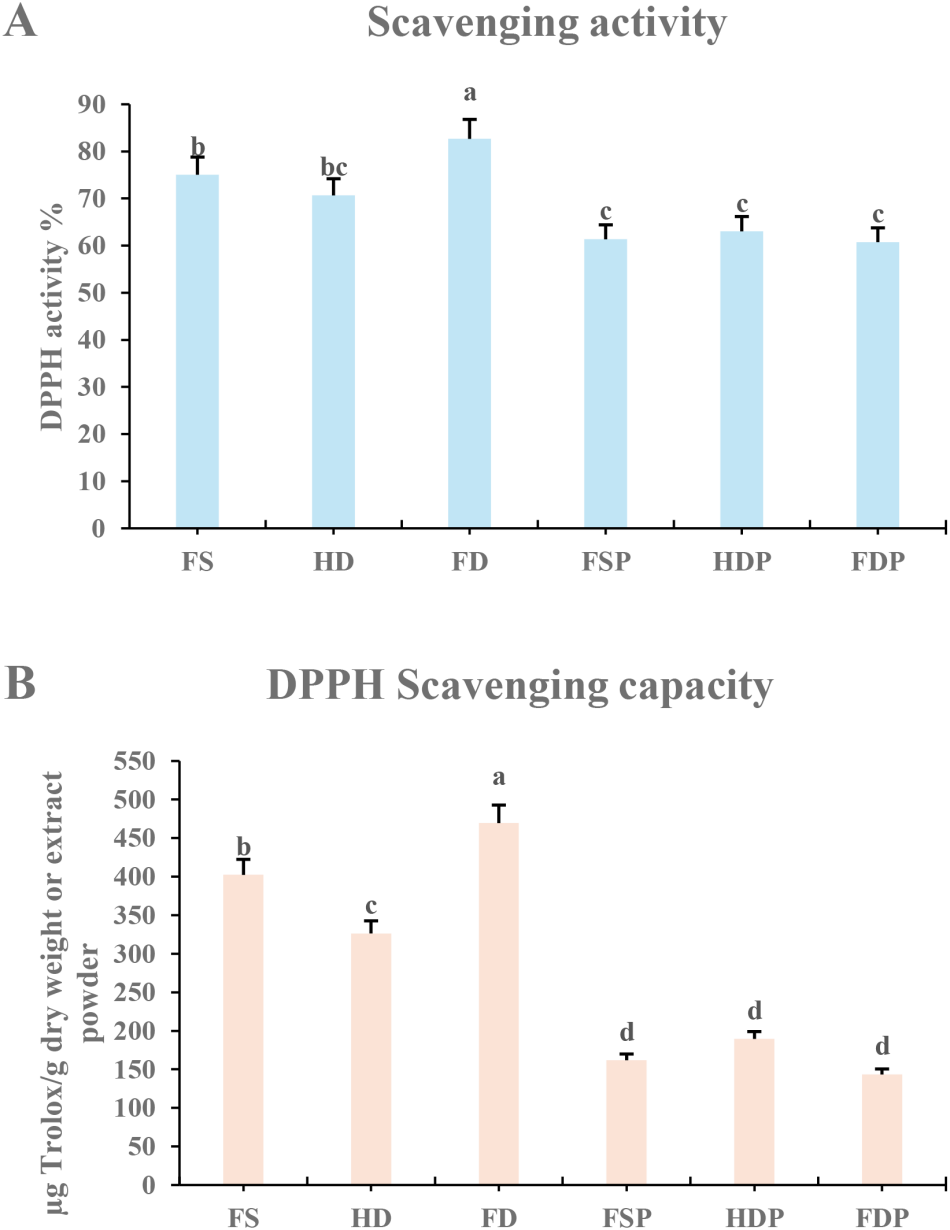
Antioxidant potential of loquat flower extracts assessed by the DPPH assay. **(A)** DPPH radical scavenging activity expressed as a percentage. **(B)** DPPH scavenging capacity quantified as Trolox equivalents. For raw materials (FS, HD, FD), values are expressed as μg Trolox per gram dry weight. For hot-water extracts (FSP, HDP, FDP), values are expressed as μg Trolox per gram extract powder. Data are presented as mean ± standard deviation (SD) of three biological replicates (n=3). Different lowercase letters above bars denote statistically significant differences (p < 0.05) among processing methods as determined by Fisher’s Least Significant Difference (LSD) test. FS, fresh sample; HD, heat-dried; FD, freeze-dried; FSP, fresh sample hot-water extract; HDP, heat-dried sample hot-water extract; FDP, freeze-dried sample hot-water extract.

The antioxidant capacity of the differently processed loquat flower extracts was quantitatively evaluated using the 2, 2-diphenyl-1-picrylhydrazyl (DPPH) radical scavenging assay. As illustrated in **Figure 6**, both the radical scavenging activity (%) and the scavenging capacity (μg Trolox/g) were significantly influenced by the processing method. Among the raw materials, the freeze-dried (FD) sample demonstrated the highest antioxidant activity (82.66%) and capacity (469.38 μg Trolox per gram dry weight), significantly higher than the fresh (FS; 75.05%, 402.28 μg Trolox per gram dry weight) and heat-dried (HD; 70.66%, 326.36 μg Trolox per gram dry weight) samples (**Figure 6A, B**). This result indicates that freeze-drying effectively preserves the antioxidant compounds present in the fresh flowers, whereas heat-drying may lead to their degradation.

A pronounced reduction in antioxidant potential was observed following hot-water extraction across all sample types (**Figure 6**). The extracts from freeze-dried (FDP), fresh (FSP), and heat-dried (HDP) flowers exhibited significantly lower DPPH scavenging activities (60.74%, 61.35%, and 63.03%, respectively) and capacities (143.39, 161.85, and 189.54 μg Trolox per gram extract powder, respectively) compared to their unextracted counterparts. The superior antioxidant activity observed in the freeze-dried sample aligns with its higher terpenoid content, suggesting that the preserved terpenoid pool, particularly triterpenoids known for their antioxidant properties, may be a significant contributor to the observed radical scavenging capacity.

## 4 Discussion

This study provides a comprehensive investigation into the impact of post-harvest processing methods, drying and hot-water extraction, on the terpenoid profile and associated antioxidant activity of loquat flowers. The application of a widely targeted metabolomics approach clearly demonstrates that processing is not a mere preservation step but a transformative process that fundamentally alters the chemical landscape of this botanical material. The most noticeable finding is that the extraction process itself exerts a more profound influence on the terpenoid profile than the specific drying technique employed, a insight with significant implications for product development in the functional food and tea industries (Koina et al., 2023; Raghunath et al., 2023).

**Figure 7:**
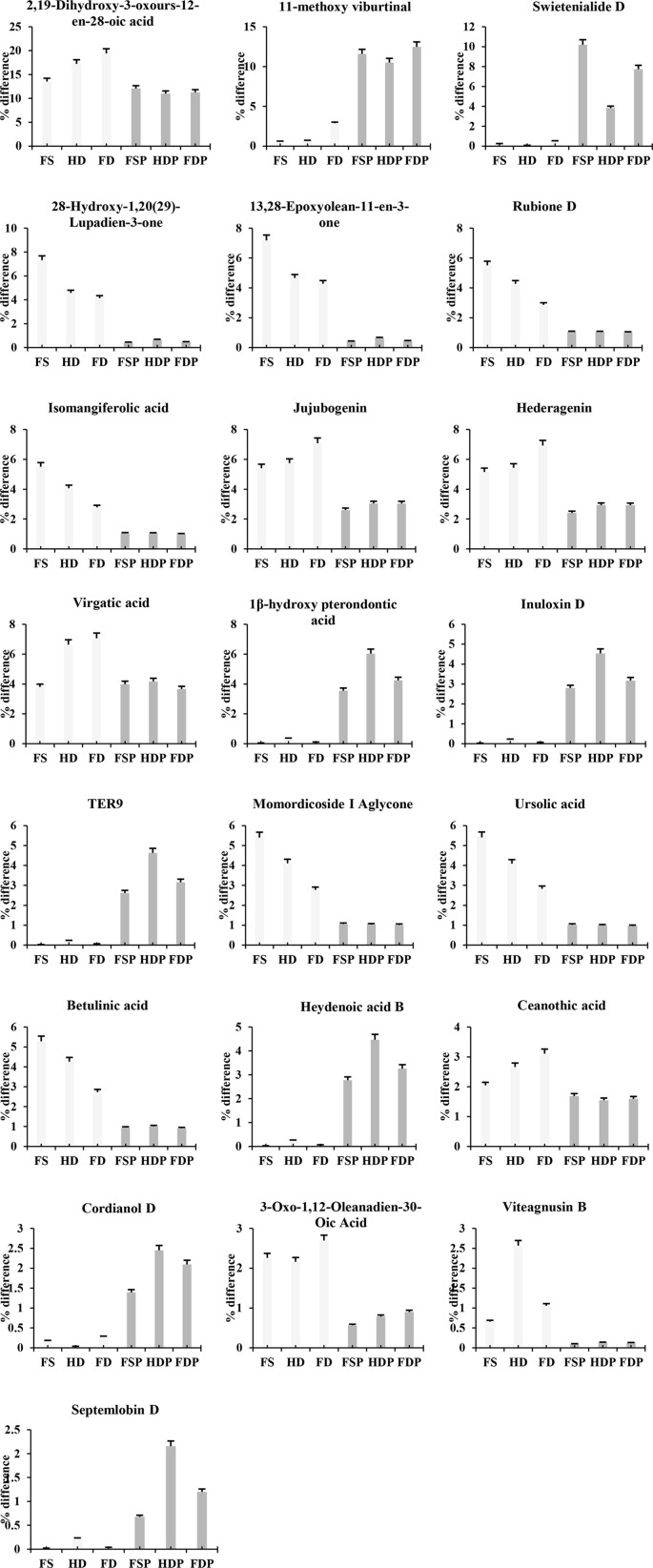
Illustrates the percentage differences in the relative abundance of 22 major terpenoid compounds across different processing methods of loquat flowers. FS, fresh sample; HD, heat-dried; FD, freeze-dried; FSP, fresh sample hot-water extract; HDP, heat-dried sample hot-water extract; FDP, freeze-dried sample hot-water extract.

Our key findings revealed a complex interplay between processing and terpenoid integrity. Freeze-drying (FD) proved superior to heat-drying (HD) in preserving the overall terpenoid content and diversity, as evidenced by the highest total peak area and the greatest number of unique compounds identified (**Figures 1** and **2**). This aligns with the well-established principle that lyophilization minimizes thermal degradation and volatilization of sensitive metabolites (ElNaker et al., 2021; ElGamal et al., 2023). The strong correlation between the terpenoid profiles of fresh (FS) and freeze-dried (FD) samples further supports this (**Figures 2**, **3**). However, the most dramatic shift occurred upon hot-water extraction. The significant reduction in total terpenoid content across all extracts (FSP, HDP, FDP), coupled with the low correlation coefficients between raw materials and their respective extracts, indicates substantial compound loss or transformation (**Figures 7**-**5**). This phenomenon can be attributed to several factors: (1) the inherently lipophilic nature of many terpenoids, leading to their poor solubility and incomplete extraction into the aqueous phase; (2) thermal degradation and dehydration of heat-sensitive compounds, particularly complex, hydroxylated triterpenoid acids (e.g., those significantly downregulated in **Table 1**), under the high temperature (90 °C) of the extraction process; and (3) potential volatilization of lower molecular weight monoterpenoids and sesquiterpenoids during both heat-drying and hot-water extraction. The superior preservation of terpenoids in freeze-dried samples aligns with the principle that lyophilization minimizes these thermal and oxidative degradation pathways (ElGamal et al., 2023).

These results directly address the identified knowledge gap by systematically quantifying how industrial processing modulates the phytochemical composition of loquat flowers (Hammed et al., 2025; Liu et al., 2025). The research question: how do drying and extraction impact the terpenoid profile? is answered with granular detail: freeze-drying best preserves the native profile, but hot-water extraction acts as a major bottleneck, creating a final product with a terpenoid signature distinct from the raw material (**Figure 7**, **Table 1**). The antioxidant activity, as determined by the DPPH assay, followed a congruent pattern (**Figure 6**). The superior scavenging capacity of the FD sample correlates with its higher terpenoid content (**Figures 1**, **6**), suggesting a significant contribution from these compounds to the overall antioxidant potential. The pronounced decline in antioxidant activity in the hot-water extracts mirrors the substantial reduction in terpenoid abundance, implying that compounds either not extracted or degraded during the extraction process, including terpenoids and potentially other antioxidants like phenolics, are key contributors to this bioactivity. This finding challenges the assumption that a simple hot-water infusion captures the full bioactive potential of the raw botanical and highlights a critical point for quality control. This correlative evidence, however, requires further validation through the direct testing of isolated terpenoids, as the contribution of other co-extracted or degraded non-terpenoid compounds cannot be ruled out. Secondly, there is a possibility that other non-terpenoid compounds (e.g., phenolics) also contribute to the overall antioxidant activity lost during extraction (Enaru et al., 2021; ElGamal et al., 2023).

Our findings are consistent with, and add a layer of specificity to, previous studies on the processing of medicinal plants. While earlier research on loquats focused on leaves or fruits, and general bioactivities (Lu et al., 2010; Cai et al., 2011; Sagar et al., 2020), this study provides a targeted metabolomic perspective on the flowers, specifically highlighting the vulnerability of terpenoids. The identification of specific terpenoids that are highly responsive to processing, such as the marked downregulation of hydroxylated triterpenoid acids (e.g., 3-O-Trans-p-Coumaroylmaslinic Acid) in extracts and the upregulation of others like 6beta, 7beta-Dihydroxykaurenoic acid, offers tangible chemical markers for optimizing processing protocols (Ye et al., 2020; Chen et al., 2024; Duan et al., 2025a; Liu et al., 2025). This moves beyond generic observations of “compound loss” to identify which specific valuable compounds are affected.

Despite these insights, this study has certain limitations. The metabolomics approach identified and relatively quantified a wide range of terpenoids, but absolute quantification for each compound was not performed. This represents an important limitation, and future work should employ authentic standards for absolute quantification of key terpenoids (e.g., ursolic acid, betulinic acid) to validate the relative intensity data and enable more precise comparisons. Furthermore, antioxidant activity was assessed using a single (DPPH) *in vitro* assay. While the DPPH assay is a valuable tool for measuring hydrogen-donating capacity, it represents only one mechanism of antioxidant action. To gain a more comprehensive and physiologically relevant understanding of the antioxidant potential, future studies should employ a complementary assay, such as the Ferric Reducing Antioxidant Power (FRAP) and Oxygen Radical Absorbance Capacity (ORAC) assays. The study also focused on a single extraction solvent (hot water) to simulate tea preparation; investigating other solvents (e.g., ethanol, hydroethanolic mixtures) common in nutraceutical extract production would broaden the applicability of the findings. Based on these findings and limitations, several avenues for future research are proposed. Firstly, absolute quantification of key bioactive terpenoids (e.g., ursolic acid, betulinic acid) across processing conditions would provide crucial data for standardizing loquat flower products. Secondly, investigating alternative extraction technologies, such as ultrasound-assisted or microwave-assisted extraction, could potentially improve the terpenoid yield and preserve antioxidant capacity, though this requires validation in future studies. Thirdly, the *in vitro* bioactivity of the significantly altered terpenoids should be validated through targeted assays to confirm their specific contributions to the observed antioxidant and potential anti-inflammatory effects. Finally, sensory analysis studies are warranted to determine how these profound chemical changes impact the flavor and aroma profile of loquat flower tea. Several terpenoids identified in this study, including linalool, α-terpineol, and pinene derivatives, which are known aroma-active compounds in various plants, likely contribute significantly to the sensory characteristics, linking the metabolomic data directly to consumer acceptance (Testa et al., 2020; Moreira et al., 2024).

In conclusion, this study establishes that post-harvest processing is a critical determinant of the final quality of loquat flower products. For industry practitioners aiming to develop loquat-based teas or supplements with maximized health benefits, the choice of freeze-drying over heat-drying is recommended to preserve the native terpenoid profile. However, the greater challenge lies in optimizing the extraction process to enhance the transfer of these valuable lipophilic compounds into the aqueous phase. This research provides a scientific foundation and a set of chemical markers to guide that optimization, ultimately supporting the creation of higher quality, standardized botanical products.

## 5 Conclusion

This study demonstrates that post-harvest processing is a critical determinant of the phytochemical integrity and bioactivity of loquat flowers. The application of a targeted metabolomics approach revealed that while the drying method significantly influences terpenoid preservation, with freeze-drying superior to heat-drying, the subsequent hot-water extraction process is the dominant factor reshaping the chemical profile. The drastic reduction in terpenoid content (69% for FSP, 59% for HDP, and 70.7% for FDP compared to their respective raw materials) and the concomitant decline in antioxidant activity in the prepared extracts highlight a major limitation of conventional preparation methods. The significant correlation between terpenoid loss and diminished DPPH radical scavenging capacity strongly suggests these compounds are key contributors to the observed bioactivity. Therefore, simply selecting an optimal drying method is insufficient; a fundamental re-evaluation of extraction parameters is imperative. These findings provide a crucial scientific foundation for the food and nutraceutical industries, emphasizing that innovative extraction strategies are essential to fully leverage the health-promoting potential of loquat flowers in functional products.

## Data Availability

The original contributions presented in the study are included in the article/**Supplementary Material**. Further inquiries can be directed to the corresponding authors.

## Supplementary material

Supplementary Table 1 Raw LC-MS data, including peak areas, compound identifiers, formulas, and masses for all detected terpenoids.

Supplementary Table 2 This table provides the abbreviated compound names used to label figures in the Hierarchical Clustering (HCA) and Principal Component (PCA) analyses.

Supplementary Table 3 Results of the statistical comparisons, including VIP scores, p-values, Log2FC, fold changes, and regulation types for all terpenoid compounds across the different treatment groups.
