# Supplementary figures and images for: Variation in terpenoids and antioxidant activity of loquat flowers during post-harvest processing: a metabolomics study
Source: Front Plant Sci. 2026 Jan 6;16:1728193. doi: 10.3389/fpls.2025.1728193 (PMC12818193; doi:10.3389/fpls.2025.1728193)

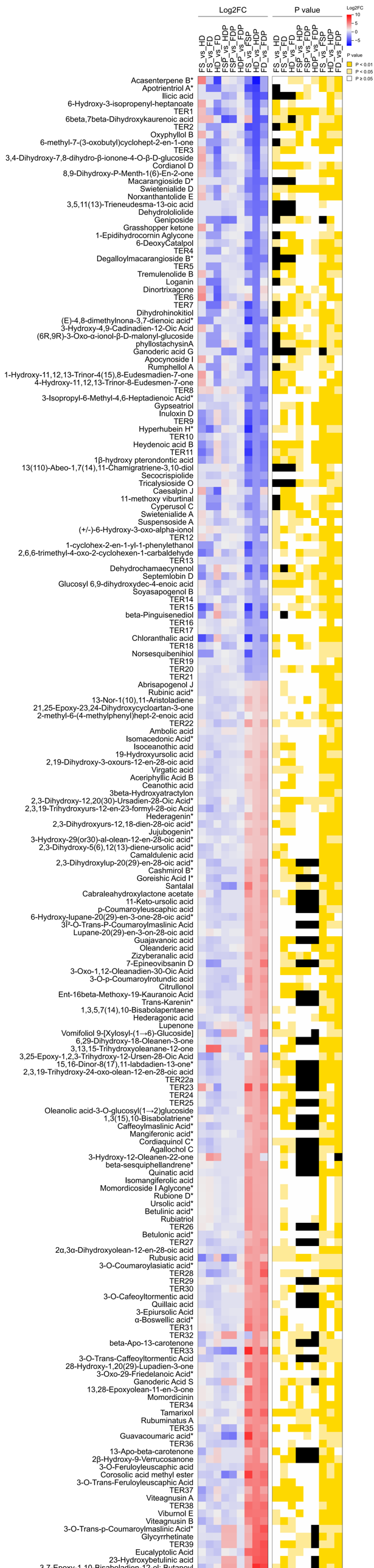

Supplement: Supplementary file 2 [file DataSheet1.pdf]
